# Austrian Lipid Consensus on the management of metabolic lipid disorders to prevent vascular complications: A joint position statement issued by eight medical societies. 2016 update

**DOI:** 10.1007/s00508-016-0993-x

**Published:** 2016-04-06

**Authors:** Hermann Toplak, Bernhard Ludvik, Monika Lechleitner, Hans Dieplinger, Bernhard Föger, Bernhard Paulweber, Thomas Weber, Bruno Watschinger, Sabine Horn, Thomas C. Wascher, Heinz Drexel, Marianne Brodmann, Ernst Pilger, Alexander Rosenkranz, Erich Pohanka, Rainer Oberbauer, Otto Traindl, Franz Xaver Roithinger, Bernhard Metzler, Hans-Peter Haring, Stefan Kiechl

**Affiliations:** Department of Internal Medicine, Medical University of Graz, Auenbruggerplatz 15, 8036 Graz, Austria; First Medical Department, Rudolfstiftung Hospital, Vienna, Austria; Medical Department, Hochzirl - Natters Hospital, Hochzirl, Austria; Department of Medical Genetics, Clinical and Molecular Pharmacology, Medical University of Innsbruck, Innsbruck, Austria; Department of Internal Medicine, Bregenz Hospital, Bregenz, Austria; First Medical Department, Paracelsus Medical University, Salzburg, Austria; First Medical Department, Hanusch-Krankenhaus, Vienna, Austria; Department of Internal Medicine and Cardiology, Feldkirch Hospital, Feldkirch, Austria; Department of Cardiology, Wels Hospital, Wels, Austria; Third Medical Department, Medical University of Vienna, Vienna, Austria; Medical Department, Linz General Hospital, Linz, Austria; Third Medical Department, Elisabethinen Hospital, Linz, Austria; First Medical Department, Mistelbach Hospital, Mistelbach, Austria; Department of Internal Medicine, Baden-Mödling Hospital, Mödling, Austria; Third Medical Department, Medical University of Innsbruck, Innsbruck, Austria; Department of Neurology, Medical University of Innsbruck, Innsbruck, Austria; First Department of Neurology, Kepler University Clinic, Linz, Austria

**Keywords:** LDL cholesterol, Vascular disease, Atherosclerosis, Primary prevention, Secondary prevention, Statin, LDL‑Cholesterin, Gefäßerkrankung, Atherosklerose, Primärprävention, Sekundärprävention, Statin

## Abstract

In 2010, eight Austrian medical societies proposed a joint position statement on the management of metabolic lipid disorders for the prevention of vascular complications. An updated and extended version of these recommendations according to the current literature is presented, referring to the primary and secondary prevention of vascular complications in adults, taking into consideration the guidelines of other societies. The “Austrian Lipid Consensus – 2016 update” provides guidance for individualized risk stratification and respective therapeutic targets, and discusses the evidence for reducing vascular endpoints with available lipid-lowering therapies. Furthermore, specific management in key patient groups is outlined, including subjects presenting with coronary, cerebrovascular, and/or peripheral atherosclerosis; diabetes mellitus and/or metabolic syndrome; nephropathy; and familial hypercholesterolemia.

## Zusammenfassung

Im Jahr 2010 haben acht österreichische Fachgesellschaften eine gemeinsame Stellungnahme zum Management von Fettstoffwechselstörungen für die Prävention vaskulärer Komplikationen erarbeitet. Für die vorliegende Neuauflage wurden diese Empfehlungen nach aktueller Studien- und Literaturlage überarbeitet und erweitert und Richtlinien anderer Fachgesellschaften berücksichtigt. Der „Österreichische Lipidkonsensus 2016“ bezieht sich auf das Lipidmanagement im Rahmen der vaskulären Primär- und Sekundärprävention bei Erwachsenen. Er gibt Anleitungen für die individuelle Risikostratifizierung und die daraus abgeleiteten Behandlungsziele und diskutiert die Evidenz hinsichtlich der Reduktion vaskulärer Endpunkte durch die verfügbaren lipidsenkenden Therapien. Des Weiteren wird auf das spezifische Lipidmanagement bei wichtigen Patientengruppen, darunter Personen mit koronarer, zerebrovaskulärer und/oder peripherer Atherosklerose, Diabetes mellitus und/oder metabolischem Syndrom, Nephropathie sowie familiärer Hypercholesterinämie eingegangen.

## Introduction

Disorders of blood lipid metabolism are well-established risk factors for atherosclerosis. The atherogenic potential of serum lipids depends on the type and concentration of plasma lipids, as well as the structure and size of lipid-transporting lipoproteins, and is also influenced by other risk markers and factors, e. g., visceral obesity, hypertension, glucose tolerance disorders and diabetes mellitus, smoking status, and genetic predispositions.

Scientific societies across the globe have acknowledged the significance of therapeutic control of dyslipidemia in an effort to prevent cardio- and cerebrovascular complications. In Austria, individual treatment recommendations have been published by, amongst others, the Austrian Stroke Society (“Österreichische Gesellschaft für Schlaganfall-Forschung”, ÖGSF; most recently in 2014) [1], the Austrian Society for Internal Angiology (“Österreichische Gesellschaft für Internistische Angiologie”, ÖGIA; 2012 and 2013) [2, 3], and the Austrian Diabetes Association (“Österreichische Diabetes Gesellschaft”, ÖDG; most recently in 2012) [4].

In 2010, eight Austrian societies issued the “Österreichischer Lipidkonsensus. Management von Fettstoffwechselstörungen zur Prävention vaskulärer Komplikationen” [5]. The present version of the consensus and its recommendations was critically revised and expanded according to the current literature. As to its contents, the “Austrian Lipid Consensus – 2016 update” is geared to the recommendations of the United States National Cholesterol Education Program Adult Treatment Panel III (NCEP ATP III; 2001, 2004) [6, 7], the recent European guidelines for the management of dyslipidemia (2011) [8] and the prevention of cardiovascular disorders (CVD; 2012) [9], and the recommendations of the European Atherosclerosis Society (EAS; 2014) [10], as well as the International Familial Hypercholesterolemia (FH) Foundation Consensus Group (2015) [11], in each case incorporating further recent study data. Additionally, the 2013 guidelines of the American College of Cardiology (ACC) and the American Heart Association (AHA) [12] are commented on.

The recommendations refer to lipid management in the framework of primary and secondary prevention in patients from the age of 18 years.

## Risk stratification

Lipid values are to be assessed within the framework of overall vascular risks. The target values and treatment strategies to be applied in individual cases are derived from the absolute risk for vascular diseases and/or complications. Drawing on the NCEP ATP III recommendations (2004 update) [7] and the joint guidelines of the European Society of Cardiology (ESC) and the EAS on management of dyslipidemias (2011) [8], cardiovascular risk is divided into four categories (low, moderate, high, very high). Tab. 1 compares these risk categories with the corresponding SCORE and Framingham risk categories [13, 14]. Basically, any of these classifications may be applied. Individual risk stratifications and corresponding treatment goals proceed along the following steps.

**Tab. 1:** Options for vascular risk assessment

Risk category	RF	SCORE^a ^(10-year risk; %)	Framingham^b ^(10-year risk; %)	Vascular and/or metabolic morbidity
Very high		≥ 10		Manifest coronary heart disease (CHD) Ischemic stroke or transitory ischemic attack (TIA) + evidence for atherosclerosis Peripheral arterial occlusive disease (PAOD) Type 2 diabetes Type 1 diabetes with end-organ damage (EOD; e. g., albuminuria) Moderate to severe nephropathy Progressive or recurrent CHD in spite of LDL‑C < 100 mg/dl
High	> 2	≥ 5	> 20	Familial hypercholesterolemia (FH) Type 1 diabetes + age > 40 years without target-organ disease Distinctly increased individual risk factors (e. g., familial hypertension, severe hypertension)
Moderate	2	1–5	10–20	
Low	0–1	< 1	(mostly < 10)	

*RF* risk factor/marker, *LDL‑C* low-density lipoprotein cholesterol

^a^ *SCORE* (Systematic COronary Risk Evaluation) is based on data from 12 European cohort studies with a total of more than 205,000 participants and gives information about the risk of cardiovascular mortality, calculated for 10 years or until age 60 [13]

^b^The Framingham tables are based on data from the Framingham Heart Study with approximately 5,000 participants and provide an estimation of absolute CHD risk over a period of 10 years (relating to the endpoints lethal/nonlethal myocardial infarction and sudden cardiac death) [14]

### Lipid diagnostics

Complete lipid profiling includes measurements of total cholesterol, high-density lipoprotein cholesterol (HDL-C), and triglycerides (TG) in the blood following a fasting period of at least 12 hours. Low-density lipoprotein cholesterol (LDL‑C) up to a TG concentration of 200 mg/dl is estimated by the Friedewald equation [15]. In the presence of TG values exceeding 200 mg/dl, it is advisable to base treatment decisions on non-HDL-C ([6, 7]; see also “Target values of LDL‑C reduction” section).

### Assessment of manifest atherosclerosis

Individuals with a high or very high vascular risk are identified by assessing the presence of diabetes mellitus, nephropathy, or of one of the following vascular disorders (see also “Specific patient groups” section):

- Coronary heart disease (CHD): status post myocardial infarction (MI) or stent/percutaneous transluminal coronary angioplasty, bypass surgery, angiographically verified CHD, ergometrically or scintigraphically proven myocardial ischemia;
- Cerebrovascular angiopathy: ischemic stroke or transitory ischemic attack (TIA) with evidence of atherosclerotic changes in the carotids, hemodynamically relevant carotid stenosis;
- Peripheral arterial occlusive disease (PAOD);
- Abdominal aortic aneurysm.

### Ascertainment of additional risk factors

Apart from lipid metabolism disorders, the following “classic” risk factors affect cardiovascular risk:

- Age (men: > 45 years; women: > 55 years);
- A positive family history of premature CHD (male first-degree relatives < 55 years; female first-degree relatives < 65 years);
- Smoking;
- Hypertension (RR > 130/80 mmHg in 24-hour measurements or > 135/85 mmHg as a mean of self-measurement, or antihypertensive medication);
- HDL-C (men: < 40 mg/dl; women: < 50 mg/dl).

A high HDL-C value is seen as a “negative” risk factor: in risk assessment, one positive risk factor should be subtracted in the presence of HDL-C values beyond 60 mg/dl.

### Risk projection and classification

- Subjects with a maximum of one classic risk factor according to the section “Ascertainment of additional risk factors” are allocated to the lowest risk category (Tab. 1).
- In individuals showing no manifest atherosclerosis according to the section “Assessment of manifest atherosclerosis”, yet with two or more risk factors according to the section “Ascertainment of additional risk factors”, risk assessments are performed with the SCORE tables [13] (or, alternatively, Framingham tables [14]).
- Subjects presenting with manifest coronary, cerebral, or peripheral atherosclerosis according to the section “Assessment of manifest atherosclerosis”, those with type 2 diabetes or type 1 diabetes and end-organ damage (EOD), and those with moderate or severe nephropathy are allocated to the group at a very high risk (Tab. 1).

### Risk-modifying factors

The following factors indicate subclinical atherosclerosis and/or EOD, or a higher risk than suggested by risk projection (“Risk projection and classification” section):

- Lipoprotein(a) (Lp[a]): > 30 mg/dl,
- Lipoprotein-associated phospholipase A2: > 200 ng/ml,
- High-sensitivity C‑reactive protein (hsCRP): > 3 mg/l,
- Hyperhomocysteinemia: > 1.6 mg/l (12 μmol/l),
- Carotid intima-media thickness: > 800 μm,
- Ankle-brachial index: < 0.9,
- Coronary calcium score: > 75th percentile,
- FH (see “Familial hypercholesterolemia” section),
- Left-ventricular hypertrophy,
- Metabolic syndrome (MS; see “Diabetes mellitus” section),
- Impaired glucose tolerance.

### Target values of LDL‑C reduction

Epidemiological studies have concordantly demonstrated a close correlation between the height of LDL‑C values and cardiovascular risk [16, 17]. Furthermore, many interventional trials have also underscored the significance of LDL‑C as a primary treatment target, whereas the benefit of lowering LDL‑C proves to be clearer with increasing total vascular risk [18, 19]. Thus for LDL‑C, incremental target and threshold values apply to the various risk categories (Tab. 2).

**Tab. 2:** Target values for LDL‑C and non-HDL-C in relation to cardiovascular risk categories

Risk category	LDL‑C target value (mg/dl)	Non-HDL-C target value^a ^(mg/dl)	LDL‑C threshold value for the initiation of medical treatment (mg/dl)
Very high	< 70^b^	< 100	70
High	< 100	< 130	100
Moderate	< 130^c^	< 160	130
Low	< 160	< 190	160

^a^Chol_non-HDL_ = Chol_total_ − Chol_HDL_

^b^And/or reduction of ≥ 50 % if the target value cannot be met

^c^In individual cases, especially in subjects with pronounced metabolic syndrome (“Diabetes mellitus” section), it may prove expedient to pursue an LDL‑C reduction to < 115 mg/dl (non-HDL-C reduction < 145 mg/dl)

Therapeutic lifestyle modification is recommended immediately after exceeding LDL‑C target values. The European recommendations for the prevention of CVD [9] comprise detailed references in terms of lifestyle modification (nutrition, physical activity) for subjects with manifest atherosclerosis or those at an increased risk of atherosclerosis. Medical intervention is indicated in subjects with low or moderate risks who exceed the threshold values in Tab. 2 after 3 months of lifestyle modification. Target value-oriented medical treatment is directly induced in the presence of high or very high risks, in an attempt to lower LDL‑C by at least 50 %.

The Friedewald equation [15] fails to provide reliable LDL‑C results with TG values beyond > 200 mg/dl. Treatment decisions in such cases should be made on the basis of non-HDL-C [6, 7], i. e., very low-density lipoprotein cholesterol and LDL‑C, including atherogenic remnant lipoproteins that are associated with hypertriglyceridemia. Post-hoc analyses of data from the TNT and IDEAL trials have shown non-HDL-C to correlate more closely with cardiovascular risk than LDL‑C [20]. Non-HDL-C target values are generally 30 mg/dl above the corresponding LDL‑C target values (Tab. 2).

## Treatment

### Medical LDL‑C reduction

The medical options to lower increased LDL‑C values include 3‑hydroxy-3-methylglutaryl-coenzyme A reductase inhibitors (statins), cholesterol absorption inhibitors (CAI), and (“old” and “new”) ion exchangers. Fibrates and niacin derivatives are second-choice drugs only in this regard.

#### Statins.

Evidence is most abundant for this drug class in terms of the reduction of cardiovascular morbidity and mortality. Large-scale interventional studies with statins have reported 27 to 35 % reductions of the relative risk for serious cardiovascular events and cardiac death [8]. A meta-analysis of primary prevention studies with a total of more than 42,800 participants resulted in a significant reduction of the relative risk for serious coronary events (−29 %), cerebrovascular events (−14 %), and revascularization procedures (−29 %) [21]. A prospective meta-analysis covering these studies and others (with a total of more than 90,000 patients) demonstrated a proportional reduction of serious vascular events in the magnitude of 21 % per LDL‑C reduction of 1 mmol/l [18]. PROVE‑IT, TNT, and IDEAL yielded a significant reduction of cardiovascular events on high-dose vs. standard statin treatment [20, 22]. Statins thus represent the first-line treatment in terms of LDL‑C reduction.

**Fig. 1:**
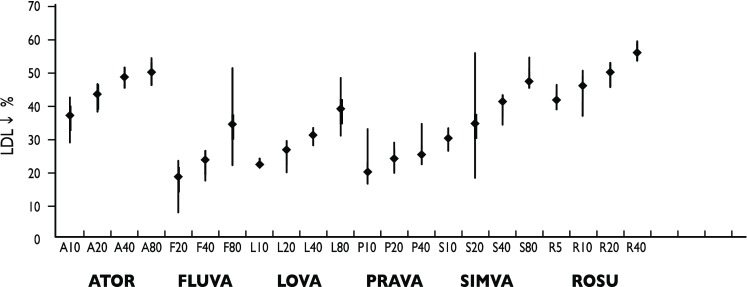
Lipid reductions effectuated with statins in clinical trials ([23], © John Wiley and Sons 2010)

Controlled studies have shown standard-dose statins—simvastatin 40 mg, atorvastatin 10 mg, fluvastatin 80 mg, lovastatin 40 mg, pravastatin 40 mg, rosuvastatin 10 mg—to effectuate placebo-cleared LDL‑C decreases amounting to 29 to 37 % of the initial values [23]. However, the interindividual differences in response are distinct. In addition, the dose–response curve is not linear, such that an additional 5 to 10 % LDL‑C reduction is to be expected with a double standard dose (an average of approximately 6 %—the “rule of six”). A placebo-subtracted LDL‑C reduction of approximately 55 % is feasible with atorvastatin or rosuvastatin at high doses (Fig. 1).

Recent meta-analyses [24–26] and population studies [27, 28] have argued that an increasing incidence of type 2 diabetes is observed on statins, especially among subjects at a higher risk of diabetes. This does not serve to change the risk–benefit assessment of applying statins in patients at a moderate or high vascular risk. A risk–benefit evaluation is to be performed in low-risk subjects.

#### Ezetimibe.

Combination studies applying the selective CAI ezetimibe together with various statins have yielded LDL‑C reductions of up to 60 % of the initial values prior to statin treatment; compared to statin monotherapy, an additional LDL‑C reduction of 4 to 27 % has been reported [29–33]. Initial endpoint data for the combination with simvastatin were produced within the framework of the SHARP study [34] in patients with chronic renal insufficiency (see “Nephropathy” section). The IMPROVE-IT study presented significant reductions in coronary endpoints, strokes, and cardiovascular deaths by additionally administering ezetimibe in patients pretreated with statins following acute coronary syndrome (ACS), in spite of low LDL‑C initial values (average 69 mg/dl [35]; see “Acute coronary syndrome” and “Cerebrovascular diseases” sections).

#### Anion exchange resins.

Anion exchange resins (AER; bile acid sequestrants) are an effective lipid-lowering option for experienced therapists. Patient tolerance has proven to be poor with older agents (e. g., cholestyramine, colestipol) on account of their side effects, with a negative influence on vitamin supply. Within this class, colesevelam has shown the most favorable side effect profile, lacking a negative influence on vitamin absorption and showing beneficial blood sugar-lowering effects [36, 37]. Colesevelam may be given as a supplement to statins or a statin-ezetimibe combination to achieve LDL‑C target values.

#### Fibrates.

Fibrates effectively address increased TG and low HDL-C values, yet decrease LDL‑C in a different way and less markedly than statins [38]. Lower rates of cardiovascular complications have been reported in subjects with MS or type 2 diabetes, and in those with low HDL-C values, although no reductions in mortality rates were reported [39, 40]. Patients with type 2 diabetes enrolled in the FIELD study and given fenofibrate experienced lower rates of MI and coronary revascularization, but no reduction in fatal coronary events or overall mortality [41]. The treatment yielded over-average benefits in participants with MS, and especially in those with severe hypertriglyceridemia [42]. In the ACCORD study, fenofibrate coadministered with statin therapy also served to reduce cardiovascular events in type 2 diabetics presenting with atherogenic dyslipidemia (high TG levels, low HDL-C), but failed to do so in the total collective [43].

The significance of fibrates in combination with statins is thus based particularly on subjects with diabetes and/or MS. Still, this combination is to be applied with care and exclusively by experienced therapists.

#### Niacin.

Niacin given alone lowers LDL‑C by 15 to 18 % as compared to initial values, with TG reductions of 20 to 40 % and dose-dependent increases in HDL-C of up to 25 % [8]. Further improvement of lipid profiles has been reported in combination with statins [44].

In contrast to older studies referring to a positive effect on cardiovascular risk in monotherapy and in combination with statins [45–47], no cardiovascular benefit was demonstrated in the AIM-HIGH study in patients with atherosclerotic vascular disease and low HDL-C who were given niacin in addition to statin [48]. In the HPS2-THRIVE study with 25,673 patients at high cardiovascular risk, a combination of niacin and laropiprant versus placebo in addition to a background statin likewise failed to reduce serious adverse events, with increases in serious non-fatal side effects in the verum group [49, 50]. Thus, the agent’s future value remains unclear [51], although niacin continues to be in international clinical use.

Following a statement by the European Medicines Agency [52], the niacin derivative acipimox, which is restricted to additional or alternative administration in lowering increased TG levels, remains the only available treatment option in this group.

#### Lomitapide.

Lomitapide inhibits the microsomal TG transfer protein, intervening in the assembly of apolipoprotein B (Apo B)-containing lipoproteins. In the presence of homozygous familial hypercholesterolemia (HoFH), this agent may reduce LDL‑C by approximately 50 %, albeit with slightly attenuating effects [53]. Lomitapide is not approved for other indications within the European Union.

#### Mipomersen.

Mipomersen reduces LDL‑C by inhibiting the synthesis of Apo B and has also demonstrated antiatherosclerotic effects in experimental investigations. However, its side effect profile limits clinical application with an LDL‑C reduction of 30 to 50 % [53]. Mipomersen has been approved for HoFH in the US, yet not Europe.

#### Monoclonal anti-PCSK9 antibodies.

Proprotein convertase subtilisin/kexin type 9 (PCSK9) elevates plasma LDL‑C levels via the inactivation of hepatic LDL receptors. Clinical phase II studies coadministering inhibitory anti-PCSK9 antibodies with statins and/or ezetimibe yielded LDL‑C reductions of up to 70 % as compared to initial values [54]. Phase III trials applying alirucumab and evolocumab and their pooled analyses have confirmed these results, additionally post-hoc and/or exploratory data demonstrated lowered rates of cardiovascular events [55–57].

Evolocumab and alirocumab were the first representatives of this class to receive approval in Europe (July 2015 and October 2015, respectively) for the treatment of patients with primary hypercholesterolemia or mixed dyslipidemias, given either alone (in the presence of statin intolerance or contraindication) or in combination with other lipid-lowering agents in addition to dietary measures.

### HDL-C and TG management

The increase of low HDL-C concentrations (< 50 mg/dl in women; < 40 mg/dl in men) and decrease of high TG concentrations (> 150 mg/dl) are secondary treatment targets. Subjects at a high and very high risk are the ones to benefit particularly from therapeutic HDL-C elevation.

Medical treatment of hypertriglyceridemia is indicated in high initial concentrations (> 500 mg/dl), whereas fasting TG levels should be lowered to at least < 400 mg/dl. This primarily serves to prevent pancreatitis; therapeutic targets in terms of cardiovascular prevention have not yet been established. Medically induced TG increases (beta blockers, corticosteroids, various psychotropics) are to be ruled out prior to treatment onset. Dietetic provisions (weight loss; restraint from alcohol, certain foodstuffs, sucrose- and fructose-containing beverages) are the basic therapeutic measures to lower TG; fibrates and the niacin derivative acipimox are most commonly used in pharmacotherapy (see “Niacin” section). Fish oil may come to be used as an alternative (in the presence of fibrate or niacin intolerance) or in combination. An intake of 3 to 4 g of fish oil per day is necessary for appropriate lipid reduction [8].

Recent studies have evidenced a direct and quantitatively underestimated connection between TG and CVD [58–61]. A therapeutic intervention against apolipoprotein CIII (ApoCIII) has been developed and resulted in a considerable reduction of ApoCIII and TG in phase II studies, soon to be approved for the treatment of patients with familial hyperchylomicronemia [62, 63]. Cardiovascular endpoint studies are under construction. Very recently, a specific Lp(a) treatment has been tested in an initial phase II study in terms of efficacy and risks [64].

### Strategies for meeting target values

Treatment choices are geared toward the lipid-lowering potency of a given agent to ensure LDL‑C decrease according to initial and target values (Tab. 2), as much as they are toward the reduction of vascular morbidity and mortality, as documented in controlled studies. Individual intolerances and contraindications are also to be given due consideration.

After exploiting lifestyle measures [9], medical treatment is typically initiated with a standard-dose statin; statin doses that are lower than those effectively used in clinical trials are usually non-expedient. Should the target value have not been met, a switch is to be made to high-dose atorvastatin or rosuvastatin according to the necessary LDL‑C reductions shown in Tab. 2 and 3. Alternatively, combination treatment may be taken into consideration. HDL-C and TG status, as well as individual tolerabilities, are to be incorporated in the given treatment decisions [8].

**Tab. 3:** LDL‑C reductions required to meet treatment targets according to initial values

LDL‑C initial value (mg/dl)	Reductions required to meet target values (%)	
	< 70 mg/dl (very high risk)	< 100 mg/dl (high risk)
> 240	> 70	> 60
200–240	65–70	50–60
170–200	60–65	40–50
150–170	55–60	35–40
130–150	45–55	25–35
110–130	35–45	10–25
90–110	22–35	< 10
70–90	> 22	–

Modified from: ESC/EAS Guidelines for the management of dyslipidaemias (2011) [8]

## Specific patient groups

The specific subsets in which metabolic lipid disorders substantially contribute to a high or very high vascular risk include manifest cardiovascular, cerebrovascular, and peripheral artery diseases; status post heart transplantation; chronic renal insufficiency; nephrotic syndrome; diabetes mellitus and MS; FH; and antiretroviral treatment in HIV infections. The following concentrates on the most crucial patient groups.

### Acute coronary syndrome

In the presence of ACS, immediate LDL‑C reduction to the treatment target of < 70 mg/dl or a reduction of at least 50 % is to be pursued—irrespective of measured lipid profiles that need not be precise in acute situations. Ideally, this value would be maintained below 70 mg/dl ([8, 65]; Tab. 2). The IMPROVE-IT study showed a significantly lower event rate in patients with ACS and a mean LDL‑C level of 54 mg/dl (simvastatin 40 mg and ezetimibe 10 mg) as compared to the control group with a mean of 70 mg/dl (simvastatin 40 mg).

### Cerebrovascular diseases

Meta-analyses of interventional trials including various vascular risk groups have generated a stroke risk decrease of approximately 22 % per 40 mg/dl (1 mmol/l) LDL‑C reduction [18, 66, 67]. In the SPARCL study [68] focusing on stroke patients without CHD, high-dose atorvastatin resulted in a significant reduction of recurrences. Based on these data and according to the NCEP Guidelines [6, 7], patients with TIA or ischemic stroke and LDL‑C values > 100 mg/dl are to be treated with lifestyle modifications, dietetic measures and a statin. Abundant arguments set out in the following speak in favor of an LDL‑C target value of < 70 mg/dl (Tab. 2):

- In line with the concept of “the lower, the better”, meta-analyses have shown a clear relationship between LDL‑C reduction in vascular patients and stroke risk [67].
- Studies directly comparing standard and aggressive LDL‑C reductions with highly potent statins have yielded a significant 16 % stroke risk reduction for the latter [67].
- In the SPARCL study, stroke and TIA patients with a LDL‑C reduction of 50 % and/or target values below 70 mg/dl experienced a risk reduction for recurrent stroke of 35 and 28 %, respectively, whereas the effect was marginal in patients with lesser LDL‑C reductions [68, 69].
- As applied in the IMPROVE-IT study, ezetimibe served to yield a significant stroke risk decrease in a collective of ACS patients whose LDL initial values already were very low (approximately 70 mg/dl) [35].
- Finally, many large-scale investigations have shown that stroke patients show an average 10-year cardiac infarction risk of clearly more than 20 %, and that they thus surpass subjects at a commonly defined high risk in terms of all relevant vascular diseases [70, 71].

Revised in 2011, the AHA guidelines accommodated these results by generally lowering the LDL‑C target value in ischemic stroke and verifiable atherosclerosis to < 70 mg/dl [72]. The dosage of statin is to be increased or a switch made to a more potent statin should the target values not be met. Statin-ezetimibe combinations may serve to meet the target values.

Strokes occurring under statin treatment are, on average, less severe and associated with an improved prognosis [73]. Discontinuation of statin therapy during the acute phase of stroke may be associated with an increased risk of death or care dependency [74, 75]. An initial, small-scale, randomized controlled study (MISTICS) has shown that the administration of simvastatin 40 mg within 12 hours post-stroke may increase the probability of clinical improvement (≥ 4 National Institutes of Health Stroke Scale, NIHSS, points after 3 days) from 18 to 47 % (p = 0.022). Whether statins may contribute to an increased risk of bleeding per se remains an open question. Data from the SPARCL study [68] and other trials are contrasting. In a recent investigation in 4,012 stroke patients undergoing intravenous thrombolysis, statins (as long-term pre-stroke medication) were not associated with an increased risk of cerebral hemorrhage [76].

The significance of medical treatments for HDL-C, TG, Lp(a), and hsCRP in the secondary prevention of stroke has not yet been established; thus, no target values can be specified for these parameters.

### Peripheral arterial occlusive disease

PAOD per se does not represent a high-risk situation that requires consequent lipid management in the absence of other risk factors (Tab. 2). Arguing in favor of a proactive approach, a recent meta-analysis showed that PAOD patients with a low initial cardiovascular risk may already profit from statin treatment (20 to 25 % reduction in vascular events) [19]. Treatment targets include delayed progression of atherosclerosis, prevention of complications in peripheral vascular surgery [77, 78] and in surgery for aortic aneurysm [79], and an overall reduction in cardiovascular morbidity and mortality [80]. Improvements in pain-free walking distance are yet another crucial benefit in many subjects [81, 82].

LDL‑C levels < 70 mg/dl or an LDL‑C reduction of ≥ 50 % are to be pursued in PAOD patients [8, 83]. To this effect, statins are to be titrated up to the maximum recommended or tolerated dose. A statin combined with an AER, with ezetimibe, or with a niacin derivative (acipimox) is to be considered should highly potent statins prove unable to meet treatment targets (Fig. 1). In the presence of statin intolerance, the ESC/EAS guidelines recommend niacin or AER. Further options include ezetimibe given alone or in combination with an AER or niacin [8].

### Diabetes mellitus

Diabetes mellitus is associated with a risk for CVD that is increased 2‑ to 3‑fold in men and 3‑ to 5‑fold in women, and is considered to be risk-equivalent to CHD [8, 84]. Irrespective of initial values, LDL‑C target values of < 70 mg/dl are indicated in patients with type 2 diabetes on account of a very high risk for vascular complications. Identical target values apply to patients with type 1 diabetes and additional EOD (e. g., microalbuminuria) [4]. The LDL‑C target value for type 1 diabetics as of age 40 years is 100 mg/dl (Tab. 2).

### Metabolic syndrome

In accordance with the NCEP/ATP-III [6, 7], MS is defined by the presence of at least three of the following criteria: fasting blood-sugar value ≥ 100 mg/dl; abdominal girth > 102 cm (men)/> 88 cm (women); serum thyroglobulin ≥ 150 mg/dl; HDL-C < 40 mg/dl (men)/< 50 mg/dl (women); RR ≥ 130/≥ 85 mmHg. As to the prediction of vascular events, this classification appears to be more appropriate than the rather pathophysiologically oriented definition [85] published by the International Diabetes Federation [86, 87].

The risk for cardiovascular events is twice as high in subjects with MS as compared to the total population [88]. As a rule, statins are the appropriate first-line treatment, although dyslipidemia in patients with MS is primarily characterized by increased TG levels and reduced HDL-C. Subgroup analyses (FIELD, ACCORD-Lipid) have emphasized the added benefit of fibrates in subjects with TG values > 200 mg/dl and/or HDL-C values < 35 mg/dl (see “Fibrates” section; [39–43, 89]).

### Nephropathy

Mildly to moderately impaired renal function may already lead to a progressive increase in cardiovascular risk in kidney patients. For this reason, various societies have recommended that chronic renal disease be classified in risk assessment as risk-equivalent to CHD [8, 90–92]. LDL‑C target values were omitted from the 2013 lipid guidelines issued by the Kidney Disease: Improving Global Outcomes (KDIGO) Group, as they failed to sufficiently predict coronary risk in patients with chronic kidney disease (CKD). Instead, the guidelines advised initiation of statin treatment as based on individual coronary risks [93, 94]. Treatment was recommended to begin based on a 10-year risk for coronary death or MI of 10 % or higher, and/or for all patients beyond age 50 years presenting with CKD stage G3 (glomerular filtration rate < 60 ml/min/1.73 m^2^) or worse, independent of the need for dialysis.

The SHARP study [34] was the first investigation to yield satisfactory endpoint data. SHARP showed patients with chronic renal insufficiency under treatment with simvastatin 20 mg plus ezetimibe 10 mg to experience a lower number of atherosclerotic events (coronary death, MI, coronary intervention, or ischemic stroke). The absolute risk reduction for this primary endpoint was 2.7 %, i. e., the number needed to treat (NNT) in order to prevent one event was 37 over 5 years. However, most of these events consisted of coronary and peripheral revascularization procedures. The absolute risk reduction in clinically relevant events, e. g., MI or coronary heart deaths, was 0.4 %, thus corresponding to an NNT of 250 subjects over 5 years. Therefore, whether or not this combination therapy is applied to a given patient presenting with renal insufficiency will be at the treating physician’s discretion. A post-hoc analysis of the IMPROVE‑IT study, additionally including several subjects up to CKD stage G3, is expected to assess the significance of statin-ezetimibe combinations in this population [35].

Neither the SHARP study nor preceding investigations (4D, AURORA) identified an effect of lipid reduction on the study endpoint in dialysis patients [95, 96]. Therefore, the 2013 KDIGO guidelines recommended to refrain from initiating, yet to continue ongoing statin treatments in this population [93]. Statin therapy reduces cardiovascular risk in patients having undergone kidney transplantation [97]. In patients presenting with immunosuppression, administration of a statin that is not metabolized via cytochrome P450 (CYP3 A) increases treatment safety. A low dose is to be administered initially and particular attention paid to side effects.

Although no endpoint studies have been carried out in nephrotic syndrome, affected subjects are to be considered as high risk and treated correspondingly.

### Familial hypercholesterolemia

In any given LDL‑C value, patients with autosomal dominant FH are at a higher vascular risk than the normal population and, in risk projection (“Risk projection and classification” section), are to be allocated to the high-risk category at least. Diagnosis may only be supported in the presence of Achilles tendon xanthoma or by genetic analysis. Specific recommendations offer details on the diagnosis and management of FH [10, 11, 98].

### Subclinical inflammation

Various studies have provided evidence that inflammatory processes contribute substantially to atherosclerosis [99]. Inflammation parameters that are measurable in serum, particularly hsCRP, can be drawn upon to improve risk stratification in various patient groups. In this respect, statins have an additional beneficial effect [100–102]. In practice, however, this does not imply an indication for lipid-lowering treatment.

## Statement on the 2013 ACC/AHA Consensus

In November 2013, the ACC and AHA published a joint guideline on cholesterol-lowering treatment [12], which deviates from previous recommendations in several cases (including the current Austrian and European guidelines [3, 4, 8, 9]).

In particular, a 30 to 50 % LDL‑C reduction or, instead, a 50 % decrease in absolute target values is called for. In addition, the introduction of a new risk score leads to a clearly expanded indication for lipid-lowering treatments in primary cardiovascular prevention. As a consequence, the benefit–risk ratio would decrease due to the lower likelihood of cardiovascular benefits with consistent risks of adverse effects (myopathy, diabetes) and the incalculable long-term consequences of statin treatment.

With respect to several patient groups, the ACC/AHA paper largely accords with the current European guidelines [8], yet the equations underlying risk assessment have not been sufficiently validated and agreed upon for European collectives. For this reason, the EAS [103] and other societies including the “D•A•CH-Gesellschaft Prävention von Herz-Kreislauf-Erkrankungen”, the AAS, and the Swiss Society of Cardiology [104] reject the positions formulated in the ACC/AHA paper.

It should be added that statins lead to interindividually highly divergent reductions in LDL‑C—an effect that, e. g., applies even more clearly to absorption inhibitors. From this perspective alone, monitoring of effectuated LDL‑C levels would seem imperative for the patients’ welfare. Since the IMPROVE-IT study additionally confirmed the concept of “the lower, the better”, low LDL‑C values in the appropriate patient groups (Tab. 1 and 2) should not merely be pursued, but also attained. A joint ESC/EAS taskforce joined this position in 2014 [105].
